# Commenting and connecting: A thematic analysis of responses to YouTube vlogs about borderline personality disorder^☆^

**DOI:** 10.1016/j.invent.2022.100540

**Published:** 2022-04-17

**Authors:** Clare M. King, Darragh McCashin

**Affiliations:** School of Psychology, Dublin City University, Glasnevin, Dublin 9, Ireland

**Keywords:** Borderline personality disorder, BPD, YouTube comments, Thematic analysis, Mental health stigma, Destigmatizing

## Abstract

Borderline Personality Disorder (BPD) is an often misunderstood and stigmatized mental health difficulty. Increasingly, social media has been used as a forum to share dialogue on such difficulties. This research analysed YouTube comments in response to personal vlogs about living with BPD. The key term ‘Living with Borderline Personality Disorder Vlog’ was inputted into a YouTube Ireland search, results were displayed by relevance and the top four vlogs that met the criteria were chosen for analysis. A total of 1197 comments (approximately 55,574 words) were analysed using inductive thematic analysis (Braun and Clarke, 2006). Five distinct themes were identified: 1) Sharing advice, support and encouragement, 2) Vlogs destigmatizing, informing and educating, 3) Solidarity, relatability and personal connection, 4) Intense, unstable intrapersonal and interpersonal functioning, 5) Prompting disclosures about mental health struggles. The vlogs gave people insight and understanding, increasing empathy towards those suffering with BPD or with their mental health. The overall picture drawn from the data was one of solidarity, support, de-stigmatization, normalization, sharing, comfort and encouragement. Further research into people's attitudes towards BPD, their opinions and knowledge of the disorder, may help make important changes, inform policies and practice, and ultimately improve the lives of those living with the disorder.

## Highlights

• This research involved a thematic analysis of 1197 comments to 4 YouTube vlogs about living with BPD.
• Five themes were identified: 1) Sharing advice, support and encouragement, 2) Vlogs destigmatizing, informing and educating, 3) Solidarity, relatability and personal connection, 4) Intense, unstable intrapersonal and interpersonal functioning, 5) Prompting disclosures about mental health struggles.
• YouTube vlogs may be utilized as an aid in peer-to-peer support for those with BPD and other mental health disorders.

## 1 Introduction

### 1.1 What is BPD?

Borderline Personality Disorder (BPD) is thought to develop in early adulthood and it is estimated that 1.6–5.9% of the population have this disorder, 75% of which are female (American Psychiatric Association [APA], 2013). There has been an increase in BPD in recent decades, especially in younger populations (Bateman and Fonagy, 2006). Diagnosis of BPD at a young age increases the likelihood for remission in the long-term, which justifies efforts towards early detection (Álvarez-Tomás et al., 2019).

Individuals with BPD may have trouble setting goals in life, struggle with intimacy and empathy, take unnecessary risks, be likely to suffer from negative moods and may have difficulty deciding who they are (APA, 2013). It is estimated that approximately 75% of people with BPD attempt suicide and 10% eventually commit suicide (Black et al., 2004). Most of those with BPD who commit suicide are under the age of 40, the majority of which are female (Paris and Zweig-Frank, 2001).

#### 1.1.1 Challenges with understanding BPD: a critique

The percentage of the population with BPD may be higher than the literature suggests as BPD is often underdiagnosed and misunderstood (Dhaliwal et al., 2020; Gunderson et al., 2018; Morgan and Zimmerman, 2015; Zimmerman, 2015). Gross et al. (2002) wrote of how ‘[…] despite the association of this disorder with suicidal ideation, comorbid psychiatric disorders, and functional impairment, BPD is largely unrecognised and untreated’ (p. 53).

BPD is often mislabelled by clinicians as bipolar disorder — despite differences in the two conditions (Gunderson et al., 2018). Compared to those with bipolar disorder, individuals with BPD have been found to be less likely to graduate from college, more likely to have other comorbid disorders, have higher levels of suicidal ideation, more frequent suicide attempts, and poorer social functioning (Zimmerman et al., 2015). BPD has been described as living in bipolar disorder's shadow — the emphasis on recognition of bipolar disorder being at the expense of enhancing the accurate diagnosis and recognition of BPD (Morgan and Zimmerman, 2015; Paris, 2004; Zimmerman et al., 2015).

BPD is highly stigmatized (Cone, 2020; Kirtley et al., 2019; Masland and Null, 2021). Stigma can become internalized by individuals with BPD (Grambal et al., 2016; Quenneville et al., 2020) and perceived BPD stigma can prevent people from accessing supports (Carrotte et al., 2019). Morgan and Zimmerman et al. (2015) suggest that reducing stigma may help improve assessment and diagnosis of BPD. More education, more public awareness and research funding may help reduce the stigma surrounding BPD diagnosis (Gunderson et al., 2018).

### 1.2 The interaction between the online environment and people experiencing BPD

There are differences to interactions in the online and offline environment, and this can have relevance for those with BPD. When communicating online, feelings of anonymity can contribute to increases in disclosures, including disclosures related to sensitive issues, including self-harm and suicide ideation (Fox et al., 2007; Misoch, 2015; Naslund et al., 2014; Suler, 2004). Being online can help increase self-acceptance in people with stigmatized identities and enable them to find likeminded people (McKenna and Bargh, 1998). Peer interactions online can have benefits for mental health recovery, as people build supportive relationships (Santesteban-Echarri et al., 2017).

In psychology, YouTube is a useful research tool (Chou et al., 2011; Khasawneh et al., 2020; Konijn et al., 2013; Miller, 2015; Möller et al., 2018). YouTube can be used to share experiences, to seek mental health information and advice, and to share first-hand experiences of mental illness (Mertan et al., 2021; Naslund et al., 2016; Woloshyn and Savage, 2020; Yoo and Kim, 2012). Naslund et al. (2014) analysed 3044 comments to 19 YouTube videos by individuals with schizophrenia (n = 4), schizoaffective disorder (n = 7) and bipolar disorder (n = 8) to examine how they related to peer-support. These researchers concluded that YouTube is rewarding for people with highly stigmatized mental illness, as they benefit from mutual learning and peer support (Naslund et al., 2014). Similarly, sharing experiences and communicating through YouTube may also bring rewards to individuals with BPD.

#### 1.2.1 Current research on BPD and the online environment

Woloshyn and Savage (2020) described video content posted on YouTube by people with BPD (n = 349) and found the content related to DSM-5 symptoms, treatment and coping methods, and hope for the future. Motivations related to gaining understanding, educating others, reducing stigma and giving others support (Woloshyn and Savage, 2020). An analysis of responder comments was beyond the parameters of the study.

Other researchers analysed 200 tweets (messages) by users on Twitter who identified with the label of BPD and found that BPD was constructed as an existence of tension and as a different existence (Dyson and Gorvin, 2017). They concluded that only individuals who have BPD can understand and connect with BPD-Tweeters (Dyson and Gorvin, 2017). A different platform (e.g., YouTube) may have elicited different results as it has been noted that Twitter is an environment that can produce negative sentiment (Dyson and Gorvin, 2017; Thelwall et al., 2011). This negativity was evident in a study by O'Dea et al. (2018) who found that 23% of replies to suicide-related tweets were dismissive or encouraging suicide. Tweets themselves are also quite restrictive- tweets were limited to 140-characters, only allowing for short messages, compared to YouTube comments that have a maximum allowed character limit of 9999 (www.techpostplus.com).

#### 1.2.2 Gaps in the current research on BPD and the online environment

Overall, there is a lack of research that focuses on analysing YouTube user comments (Miller, 2015). YouTube comments may be understudied as they are vast, lack structure, and have great variability in vocabulary and grammar (Madden et al., 2013). However, comments are likely to be more spontaneous, authentic, and not as heavily edited as the YouTube videos themselves — often offering genuine personal reactions (Miller, 2015).

People with mental health issues (particularly those with schizophrenia, bipolar disorder, major depressive disorder and schizoaffective disorder) are more likely than the general population to engage in vlogging (Sangeorzan et al., 2018). Sharing lived-experiences can promote user interaction which, in turn, can help combat stigmatization (Tomas et al., 2015). Vlogging can provide an individual with peer support, enhance self-efficacy, reduce self-stigma and encourage recovery (Sangeorzan et al., 2018). Comments to vlogs can give access to a wide variety of opinions and responses, however there is a lack of research into the comments to BPD-centred vlogs, about a disorder that is often stigmatized, underdiagnosed and misunderstood (Carrotte et al., 2019; Dhaliwal et al., 2020; Zimmerman, 2015).

### 1.3 Aims

The aim of this research is to gain insight into how YouTube commenters are responding to BPD-centred vlogs, an area that has not yet been analysed. The comments are the focus of this study. The comments are written responses to vlogs and include information on a range of topics, posted by various YouTube users. Naslund et al. (2014) analysed YouTube comments to videos about individuals with other mental health disorders, however there is no similar research on comments to YouTube vlogs or videos about BPD. YouTube video content posted by people with BPD has been analysed (Woloshyn and Savage, 2020), as have tweets by people identifying as having BPD (Dyson and Gorvin, 2017). However, this current research is different as it focuses on YouTube user comments to vlogs, i.e., on the responders- the commenters. Specifically, the researchers aim to gain insight into people's attitudes towards and opinions about BPD, their knowledge of the disorder, and what responses the vlogs prompt.

## 2 Method

### 2.1 Participants

The participants were YouTube users that had commented on either of the four top vlogs that appeared on the listed results when searching ‘Living with Borderline Personality Disorder Vlog’ on YouTube. Comments to vlogs can only be made by people with a YouTube account or a Google+ account. All participants should be over 18 years of age. YouTube stipulates that young children cannot post and that teenagers require parental consent.

### 2.2 Materials

The YouTube Ireland public platform was used to search for and gather the data. The Patient Education Materials Assessment Tool for Audio-visual Materials (PEMAT-A/V) was used to indicate the quality of the video content (Appendix A; Shoemaker et al., 2014). This 17-item checklist allowed for calculation of an understandability score and an actionability score: the higher the understandability score the more understandable the material is, the more people can process and explain the key messages (Shoemaker et al., 2014). The higher the actionability score the more people can identify what they can do based on the information given (Shoemaker et al., 2014). The PEMAT-A/V is a reliable and valid measure of understandability and actionability of audio-visual materials with strong internal consistency (a = 0.71) and it has been through consumer testing to establish construct validity (Shoemaker et al., 2014). It was specifically developed for audio-visual materials on diverse topics and allows for assessment of materials themselves, with no other information (Shoemaker et al., 2014). This is of importance as information on the vloggers themselves (e.g., their motivations and information) may not be readily available and is outside the scope of this research. The PEMAT-AV can not only help to judge the quality of materials about mental health issues but also benefits from ease of use, as it does not require training and can even be used by the general public (Shoemaker et al., 2014). Comments were initially copied and pasted into Microsoft Word, then after the removal of unsuitable comments (i.e., blank or offensive), were transferred onto NVivo software for analysis.

### 2.3 Procedure

The research went through an ethics review process (DCU, Psychology Ethics Committee [PEC]) and received ethical approval (ethics approval number DCUPEC_2021_161). There is a risk that a participant/YouTube commenter becomes aware that their words, their comments, have been used in this research. This might cause them upset, psychological distress or embarrassment. To manage this risk, the researchers had an ethical obligation to not cast judgement upon the users and to remain objective throughout. The research adhered to the British Psychological Society's Guidelines for Internet-Mediated Research of Respect for the Autonomy, Privacy and Dignity of Individuals and Communities, Scientific Integrity, Social Responsibility and Maximising Benefits and Minimising Harm (British Psychological Society, 2021). To select the vlogs, the key term ‘Living with Borderline Personality Disorder vlog’ was inputted into a YouTube Ireland search. The resulting vlogs were displayed by relevance, an option on YouTube that ranks in descending order the videos that best match the search term and have driven engagement. The top vlogs selected have higher view counts, which also tend to have higher numbers of comments (Miller, 2018). The inclusion criteria for the vlogs was that 1) they were related to BPD and had BPD in the title, 2) that they were in English, 3) that they had comments enabled, and 4) were posted by adults over 18. The top four vlogs that met the criteria were chosen for analysis and their quality was assessed using the PEMAT-A/V (Shoemaker et al., 2014).

As some YouTube videos have thousands of comments, only a subset of comments were used, otherwise the research would have been overly cumbersome (Miller, 2018). The *Top Comments* are sorted by YouTube based on how many users like versus dislike a comment (Khasawneh et al., 2020). The equivalent of the first five pages of comments – the first 375 comments – or all the comments for each vlog were collected for analysis, whatever number was lower (Möller et al., 2018). The comments were copied and pasted into Microsoft Word. Inclusion criteria for the comments were that 1) they had to be in English and 2) they had to include words. Also, spam, or comments that were incomprehensible, vulgar, or offensive were disregarded, as were any comments deemed to be from anyone who declared that they were under 18 years of age (Miller, 2015). Comments were transferred to NVivo. Comments were analysed using accessible and theoretically flexible inductive thematic analysis (Braun and Clarke, 2006).

The comments were handled with sensitivity and the comments were analysed, not the commenters. Every effort was taken to protect the identity of the commenters: in the findings, no usernames were given and, as has been identified as best practice, comments were paraphrased, so there are no word for word direct quotes in the final report (Miller, 2018; Reilly, 2014; Khasawneh et al., 2020). When paraphrasing, the meaning was not changed (Khasawneh et al., 2020). Paraphrasing helps to protect the identity of the user and allows the researcher to fix any inaccuracy or typographical error (Khasawneh et al., 2020).

### 2.4 Data analysis

Vlogs and comments were taken from YouTube and were publicly available (Miller, 2018). The vlogs were described and categorised using the PEMAT-A/V assessment tool (Shoemaker et al., 2014). The comments were transcribed and analysed using inductive thematic analysis (Braun and Clarke, 2006, Braun and Clarke, 2021). The analysis followed the six phases as established by Braun and Clarke, 2006, Braun and Clarke, 2021. *Phase 1: Familiarizing yourself with the data:* All of the comments were read as they were extracted from YouTube, read and re-read in the Microsoft Word review and initial ideas noted. *Phase 2: Generating initial codes*: The coding was data driven, the researcher worked systematically through all comments, giving each item attention. *Phase 3: Searching for themes:* This was the beginning of analysis, with the codes collated into potential themes. *Phase 4: Reviewing themes:* The themes were refined, being sure that they worked in relation to the codes and the data set as a whole. *Phase 5: Defining and naming themes*: There was ongoing analysis with the essence of each theme being identified, giving clear names and definitions. *Phase 6: Producing a report:* This involved telling the story of the data and providing evidence of the themes using explanations and examples (Braun and Clarke, 2006, Braun and Clarke, 2021).

## 3 Results

The four top vlog results of the YouTube search (‘Living with Borderline Personality Disorder vlog’) met the inclusion criteria. All vlogs had been uploaded by female YouTube users who identified as having BPD. The four vlogs had an average of 239,893 views (from 42,460 to 709,340; see Table 1). The vlogs had a combined PEMAT-A/V understandability score of 68.4% and a combined PEMAT-A/V actionability score of 50%. After the review of comments, 27 comments were deleted and the remaining 1197 comments (approximately 55,574 words) were then transferred to NVivo for analysis (see Table 1).

**Table 1:** Vlog and comment details.

Data	Vlog 1	Vlog 2	Vlog 3	Vlog 4	Combined total
Total comments available	319	6955	339	191	7804
Comments	8	0	13	6	27
Removed in review	(8 = emoji only, no words)		(7 = no words; 1 = offensive; 1 = spam; 1 = incomprehensible; 3 = not in English)	(1 = no words; 3 = incomprehensible; 2 = offensive)	
Comments analysed	311	375	326	185	1197
Word Count of Comments Analysed (Approx.)	11,900 (average comment length 38 words)	19,742 (average comment length 53 words)	12,441 (average comment length 38 words)	11,492 (average comment length 62 words)	55,574 (average comment length 46 words)
Views	48,765	709,340	158,708	42,760	959,573
Length	30 min 07 s	17 min 23 s	18 min 59 s	29 min 10 s	95 min 39 s
PEMAT understandability score	70%	78%	80%	44%	68.4%
PEMAT actionability score	67%	67%	33.3%	33.3%	50%
Date vlog posted	03/05/2021	02/02/2019	17/03/2019	23/10/2019	

After analysis of 1197 comments, five distinct themes were identified: 1) Sharing advice, support and encouragement, 2) Vlogs destigmatizing, informing and educating, 3) Solidarity, relatability and personal connection, 4) Intense, unstable intrapersonal and interpersonal functioning, and 5) Prompting disclosures about mental health struggles.

### 3.1 Theme 1: Sharing advice, support and encouragement

Sharing advice, support and encouragement was a main theme. The vloggers were a source of advice and guidance for many: called inspirations, role models, and looked up to as big sisters. They were a source for seeking information and advice, evidenced by the questions people asked them such as requesting more information on how to get a BPD diagnosis, how to tell if someone has BPD, its symptoms, causes, how to cope and how to help others that have or may have BPD. Vloggers were also asked about their experiences of medication, how they cope with their emotions, how to find and approach a therapist, and questioned about the effectiveness of mindfulness and the availability of online group therapy. Some commenters wrote of how they were learning about BPD and about themselves, or about others, through the vloggers personal stories. They wanted to learn more about the vloggers experiences of disassociation and self-harm, more about their childhood, more about their partners' points of view, and wanted more vlogs about the same subject.

The vloggers were inspiring people to keep going, giving many hope that they could manage their symptoms, get through their diagnosis, that they could cope. The vloggers were helping many figure things out and many commenters wrote of gaining insight into themselves through the vloggers stories. Some commenters explained how they were afraid to get help but that hearing the vloggers' experiences made them change their minds, made them realize therapy could help them too. The vloggers were described as having encouraged many commenters to see a therapist, make an appointment with a psychologist or to seek a diagnosis. Overall, many commenters wrote of learning from the vloggers openness, honesty and transparency.

The vloggers were a source of support and encouragement as they calmed many people, made them feel less alone, worthy, stronger, better. This support was described as needed, and many commented that it had come at just the right time: that they had needed to hear it now, today, tonight. With support from the vloggers, many people felt more confident in opening up themselves and sharing their feelings — which they did through the comments. The vloggers courage was credited by many for encouraging them to share their own BPD diagnosis with family and friends. The vloggers motivated some commenters to work out, look after their mental and physical health, and to move forward in the dark times. Importantly, the vloggers gave many people who had mental health issues hope: for example, hope that they could achieve their dreams and goals, gain control, be successful, find someone to love, and have a fulfilling life — a good life.

It was not just the vloggers but many of the commenters themselves who gave guidance and advice. They discussed what they thought caused BPD and how best to deal with it. The main causes commenters identified were trauma and stress, with some advising the vloggers that they may have a different diagnosis, such as PTSD. Many commenters gave advice by sharing how they coped with BPD and mental illness themselves. Various strategies were put forward by different commenters to help those suffering with their mental health such as acupuncture, mantras, affirmations, fitness, Buddhism, religious faith, distraction and grounding, EFT tapping, mindfulness, being in nature, talking about repressed emotions and seeking early intervention. The treatment methods for BPD advised most by commenters were Dialectical Behavioural Therapy (DBT), meditation and journaling. Some commenters advised that acceptance is important, acceptance of the self, self-worth, and acceptance from others. The vloggers themselves were given various pieces of advice such as to go easy on themselves, take time to achieve their goals, to concentrate on breathing when overwhelmed, to ignore criticism and never give up. Some commenters gave words of support and encouragement to all with BPD, some wrote how people with BPD could be happy, others wrote of how BPD can be treated, conquered, that people could recover from it, that those with BPD are valid human beings who deserve to be treated well. Many commenters sent love to all people with BPD or a mental health issue, and some commenters wrote that they were praying for those with BPD and sending them blessings.

The vloggers were admired by many for sharing their stories, for their authenticity, and for being genuine, for being real and open. There was a sense of loyalty and encouragement from most of the commenters, with many writing of how the vloggers were strong, brave and courageous, many were proud of them (and some had been followers of the vloggers for years). Each of the vloggers had their appearance praised: the vloggers looked good, were beautiful, pretty, hot, gorgeous, had pretty hair and nice clothes, with a few commenters expressing their surprise that someone so attractive could have something in common with them (a mental health disorder). There were many words of congratulations given to the vloggers: various commenters offered the vloggers congratulations for their accomplishments and for how far they had come, for their success, for keeping going, and for posting and sharing the vlog. The vloggers received well wishes and messages of support from many commenters who claimed that they had BPD or a mental health condition and also support from many commenters who claimed they didn't have a mental health issue. There was a strong sense of encouragement, as vloggers received many comments that told them to either keep going, get stronger, keep fighting, stay strong, keep pushing, stay beautiful, keep their head up, never give up, keep living, and most frequently, were asked to keep sharing their story.

### 3.2 Theme 2: Vlogs destigmatizing, informing and educating

The vlogs were an important force for destigmatizing BPD and mental health issues and the vloggers themselves were praised by many commenters as being advocates for mental health and for those with BPD. Through the comments, it was clear that BPD is a disorder that needs to be talked about more, as many commenters explained how the disorder was stigmatized. Some commenters wrote of how mental health issues have a negative stigma but the vloggers – through their courage, bravery and transparency – were helping to end that stigma. It was claimed by many commenters that the vlogs were normalizing BPD and mental health issues. The vlogs were reassuring many of the commenters who claimed to have BPD or a mental health illness themselves. Normalizing mental illness made some people feel like less of bad person, some wrote of feeling less crazy, others wrote of feeling less afraid of their new diagnosis of BPD, and many wrote of feeling validated. Some described being comforted and others empowered by the de-stigmatization: writing of how it had a ripple effect, encouraging them to talk about their BPD or mental health issues with friends and family. However, not all commenters felt empowered by the vlogs. Some admitted that they were afraid to talk about their own BPD, that they could never publish a vlog describing their own experiences.

The vlogs were described by many as being important as they gave representation to those with BPD and with mental health issues (there were frequent references made to how the vloggers described BPD perfectly). Many of the commenters that disclosed their own BPD diagnosis wrote of the vlogs making them feel understood, or seen and heard, and many wrote of vloggers as having given them a voice. The vloggers were looked on by many commenters as BPD advocates and heroes, praised for articulating what many commenters were feeling (for some, describing feelings that they could not articulate themselves). Vloggers were praised often for giving great explanations and some commenters wrote of how they were going to show the vlog to their friends and family so they could better understand them.

The vlogs were praised and complimented by many as helpful, positive, and progressive. Many commenters wrote of how the vlog they watched left them amazed, that it was wonderful, or that it was useful and well done. Some of the commenters described their difficulties in trying to find information about BPD online and wrote of how the vloggers explained it in a way that they could understand. Some commenters claimed they had similar symptoms to what was described in the vlog and wrote of how – after watching the vlog – they felt that they had finally gotten some answers. The vlogs made some people realize they might have BPD and for some, the explanation was a relief. There were commenters who wrote of having lived their whole lives feeling different but not knowing what it was. After watching the vlogs, some people were left questioning previous diagnoses that they had received from clinicians, suspecting that they were misdiagnosed with something else – namely depression, anxiety, an eating disorder or bipolar disorder – when it was really BPD. After watching the vlogs, some people felt the vloggers' symptoms were similar to their loved ones. Overall, the vlogs gave many people insight and understanding, increased empathy in many towards those suffering with BPD or mental health issues, and increased awareness of many of the commenters own mental health.

A testament to the vlogs usefulness as a tool for informing and educating is the gratitude expressed to the vloggers: the vast majority of commenters thanked the vloggers, expressing how grateful and appreciative they were. The vlogs prompted some to go to extra efforts to thank them, as some commenters explained that they rarely commented on YouTube but felt that they had to comment on the vlog to express their appreciation. Honesty, openness and transparency with the topic was hugely important to many commenters. People who disclosed their professions as clinicians, social workers, nurses and therapists, were among those grateful to the vloggers for their honesty and courage, for highlighting and discussing mental health, destigmatizing BPD and providing BPD psychoeducation. There are many expressions of gratitude to the vloggers for discussing, normalizing, and helping to break the stigma of mental health illness. Information and knowledge about BPD proved to be hugely important for many people, with many grateful because the vlogs changed their lives: helping them pull themselves out of a pit of despair, helping them when they thought all hope was lost, when they were going to give up, and some commenters even wrote that the vlog literally saved their lives.

### 3.3 Theme 3: Solidarity, relatability and personal connection

The vloggers established a bond with many of the commenters, a solidarity was created between them, fuelled by high relatability to the vloggers stories, conveyed in a way that made many commenters feel they were right there with the vloggers and that they were in this together. Some commenters invited the vloggers to direct message them for a chat or support. Many commenters stated that they felt less alone (or not alone) because of the vloggers, and many commenters also reassured the vloggers that *they* were no longer alone. There was also a solidarity between many of the commenters who claimed to have BPD and the vloggers. Some commenters explained that people with BPD supported each other and understood each other, others described themselves as a big family, who care about each other.

What the vloggers spoke about – their emotions, thoughts, experiences, and behaviours – proved highly relatable. Many commenters felt that the vloggers were the same as them, like they were watching themselves and describing their life. Many commenters had experienced the same things as the vloggers, agreed with what the vloggers said, and the similarities gave some commenters goosebumps. Examples of what the commenters were relating to include the need for constant reassurance, experience of obsessing over people, feeling like they had no control and finding other people difficult to get on with or be around. Some even saw similarities between themselves and how the vloggers spoke, fidgeted, or gestured. Some also wrote of experiencing trouble in school, being bullied, having difficulties in relationships and cutting off ties with people – just like the vloggers. Experiences were also disclosed, such as panic attacks and dissociative episodes, as a way of showing the vlogger that the commenters understood them. Many described having similar symptoms to the vloggers, including self-harm, hyper-sensitivity, pain and anger, trying to get a sense-of-self from others and fear of abandonment.

This high relatability to what the vloggers were saying helped to create in many a feeling of personal connection and closeness to the vloggers. The connection between commenters and vloggers was emotional, filled with empathy. Many commenters wrote of feeling the vloggers' pain, of crying for the vloggers or of crying with the vloggers as they watched the vlogs. There were expressions of feeling like the vlogger was a friend, or requests to become the vlogger's friend. Many commenters wanted to know the vloggers in real life: there were requests to meet the vloggers, talk to or chat with the vloggers. There were many declarations of love for the vloggers and expressions of wanting to hug them.

### 3.4 Theme 4: Intense, unstable intrapersonal and interpersonal functioning

When describing their symptoms and struggles with mental health, many commenters were affected by intense and unstable intrapersonal functioning or by intense and unstable emotions, reactions, thoughts and/or sense of self. Many of these commenters described finding it hard to regulate and control emotions — emotions that some found hard to articulate. Mood swings that caused emotional highs and lows were described by some as akin to an emotional rollercoaster. Intense emotions, such as feelings of intense rage and anger to feelings of devastation and numbness, would leave some commenters feeling drained. Some commenters explained that they could feel extreme guilt and shame over having a mental illness. Other commenters wrote about having extreme sensitivity to others, explaining how a perceived slight could trigger an emotional outburst or overreaction, examples of which included cutting people off then regretting it, self-harming, losing it, being unable to calm down, getting furious and blowing the tiniest of things out of proportion. Some commenters explained that in hindsight, they would realize their reactions were extreme but that they couldn't control themselves. Many commenters also wrote of how they struggled with over-thinking. They had intense, obsessive thoughts, often about others, that they could not control. These thoughts were described as intense, negative, and intrusive, making it hard to think rationally and, in some cases, leading to disassociation or thoughts of suicide. These intense and unstable emotions and thoughts were compounded by difficulties with establishing a sense of self, with some commenters not feeling like they had a true self, and others not understanding themselves or their personality. An unstable sense of self meant that at times some commenters would not recognise themselves or would feel like someone else had taken over their body. Some commenters explained how these issues could lead to them regularly changing their appearance and self-image or pretending to be someone else.

Many commenters were affected by intense and unstable interpersonal functioning, manifesting in many as difficulties with others and sabotaging relationships. Despite wanting friends, wanting love and wanting relationships, many commenters wrote of finding it hard to make friends and hard to keep them. Many commenters explained how they were afraid that others would abandon or reject them, and this is why many preferred to be alone. These commenters had experienced tumultuous relationships with other people and had been hurt by others. Many of those who claimed to have BPD had been shunned by their families, been vilified by others, and called names such as overdramatic and attention seekers. A lot of this hurt by others was claimed to be caused by a lack of others understanding of BPD and because BPD carried a stigma. However, this hurt appeared to go both ways as other commenters described being hurt by either a family member, spouse, child, parent, friend or partner with BPD, some being pushed away and villainized. The disorder was blamed by some for the breakdown of marriages or estrangement from family members. Some commenters wrote of how it could be emotionally exhausting and hard to watch someone with BPD suffer. At a loss, some of these commenters asked for advice on how to deal with being around someone with BPD and on how to help them.

Functioning in the world was unstable for some commenters, as they wrote of being unsure of what they wanted out of life and of struggling setting and reaching goals. Some described how they would watch other friends progressing in life, graduating college, getting jobs, getting married, while they felt stuck. With difficulty functioning, these commenters described how they struggled to complete school, skipping classes, taking a year off or dropping out. These commenters also wrote how they struggled with jobs: quitting jobs, changing jobs often, or unable to keep a job. It was speculated that these struggles in life were because of extreme mood swings, lack of motivation and feelings of dread.

### 3.5 Theme 5: Prompting disclosures about mental health struggles

The vlogs prompted disclosures about experiences of mental health struggles as many viewers opened up in the comments, sharing personal details. A lot of commenters spoke about their experiences of a number of disorders: Attention Deficit Disorder (ADD), Generalised Anxiety Disorder (GAD), Social Anxiety Disorder, BPD, Bipolar Disorder, eating disorders, Obsessive Compulsive Disorder (OCD) and Post-Traumatic Stress Disorder (PTSD). Many commenters wrote of how either they themselves, or their family members or friends, suffered from these disorders. Many of the commenters who claimed to have a BPD diagnosis wrote of it as being a complex and frightening disorder, that it was hard – and for some even hell – to live with. Some commenters wrote of having BPD since they were teenagers and of how they were still suffering from the disorder in mid-late adulthood. Some also wrote of feeling that their BPD symptoms were getting better but then relapsing and feeling like they were going round in a constant circle.

Aside from BPD, the most common mental health disorder disclosed was depression, with many commenters disclosing their daily battle with depression, or of how it could leave them feeling empty with low self-worth or suicidal thoughts. It was thought by some that mental health disorders had stemmed from trauma or abuse in childhood and some opened up about trying to come to terms with this trauma and about trying to stop repressing painful memories.

There was no consensus on treatment approach or on whether recovery was possible. It came to light that many found therapy too expensive so went without it, and some had tried therapists but didn't feel they were a good fit for them, or thought therapists misunderstood them or misdiagnosed them. The use of medication had mixed effects and was endorsed by some and condemned by others. For example, one wrote that medication stopped them from hitting rock bottom, others wrote of how it stabilized them and helped them to get through the day, another commenter wrote of how medication made them get their life back. However, others wrote of medication making them numb, others described the years they took medication as lost, and one commenter claimed medication caused hormone imbalances. Whether for or against, there was a resigned acknowledgement from some commenters that some people would be on medication for life. There was also agreement from many that medication alone was not the answer. The support of others was identified by many as a decisive factor in coping with a mental health issue, be it family members or a partner. Unfortunately, many who claimed to suffer from a mental health condition wrote of how they were embarrassed, or afraid to ask for help, and one commenter was absolutely horrified by their BPD diagnosis.

It was common for those with mental health issues to be hard on themselves or to think of themselves as a bad person. Though a struggle, some saw their mental health issues as a blessing as they made them stronger, more resilient or have more empathy for others. They wrote of how mental illness could be recovered from, get better with time, could change- could, as one commenter wrote, at least be bearable.

## 4 Discussion

The objective of this research was to gain insight into how YouTube commenters are responding to BPD-centred vlogs. After thematic analysis, five main themes were identified: 1) Sharing advice, support and encouragement, 2) Vlogs destigmatizing, informing and educating, 3) Solidarity, relatability and personal connection, 4) Intense, unstable intrapersonal and interpersonal functioning, and 5) Prompting disclosures about mental health struggles. The themes indicated that commenters and vloggers were creating networks of support. Feelings of personal closeness appeared to create a bond of trust between them, encouraging disclosures and the sharing of stories. There was also a unity in some of the opposites running throughout the comments: a strength in vulnerability, a togetherness from talking about being alone. The vloggers were not just informing and educating, but in doing so, were also comforting, changing – and even saving – lives. The vlogs themselves are destigmatising BPD and encouraging others to talk about their mental health. People wrote how they gained a new or deeper understanding about BPD and with that, an increased empathy and support for those suffering from BPD and other mental health issues.

### 4.1 YouTube vlogs as a source of support

Just as YouTube videos about schizophrenia, schizoaffective disorder and bipolar disorder have benefits for commenters (Naslund et al., 2014), so too did the YouTube vlogs about BPD. The overall picture drawn from the data was one of solidarity, support, de-stigmatization, normalization, sharing, comfort and encouragement. These YouTube findings are in contrast to those from studies on Twitter: with BPD-Tweeters viewing themselves in opposition to others, believing that no one could understand or connect with them (Dyson and Gorvin, 2017). The YouTube commenters (both with mental health issues and without) responded to YouTube vlogs about BPD with empathy, understanding, gratitude, relatability and a personal closeness. It appears that although Twitter may be an environment that produces negative sentiment (Thelwall et al., 2011), YouTube may be a platform that encourages more positive sentiment from commenters.

### 4.2 Mental health stigma and lack of knowledge

The vlogs were destigmatizing, informing and educating others on BPD, a disorder that is highly stigmatized and misunderstood (Cone, 2020; Kirtley et al., 2019; Masland and Null, 2021; Zimmerman et al., 2015). Researchers Thomas et al. (2015) have highlighted the need for further research into the utilization of online interventions in reducing stigma – and self-stigma – for those suffering with mental health issues. The use of vlogs to educate, raise awareness and reduce stigma (Cunningham and Craig, 2017; Gunderson et al., 2018) may be particularly beneficial, as greater numbers of people are turning to YouTube for advice about mental health (Naslund et al., 2016). The vloggers were questioned on a variety of issues related to BPD and these questions evidence the lack of knowledge on BPD and the desire to know more. The variety of different treatment approaches suggested and experienced by many commenters who claimed to have BPD demonstrate the lack of consensus on what works best. There appeared to be a self-stigma to having BPD, with the vloggers perceived as brave and courageous for talking about a disorder with such a bad reputation. People with BPD have been shown to have higher levels of internalized stigma than other disorders (Grambal et al., 2016; Quenneville et al., 2020). Even talking about mental health can be stigmatized — though the commenters claim that by sharing their stories, the vloggers are helping to overcome that stigma.

### 4.3 Clinical implications: underdiagnosis of BPD and utilization of peer-to-peer supports

Researchers have found that BPD can go largely undiagnosed (Gross et al., 2002). Many commenters suspected that they had BPD. Commenters also wrote about experiences of misdiagnosis: of having their diagnosis changed to BPD years after an initial diagnosis of bipolar or depression. Researchers have found that BPD is often mislabelled (Gunderson et al., 2018) and it has been suggested that there has been an emphasis on the recognition of bipolar disorder at the expense of accurate diagnosis of BPD (Morgan and Zimmerman, 2015; Zimmerman et al., 2015). These findings have clinical implications as delays to diagnosis may affect treatment outcomes and further perpetuate BPD stigma. An improvement of assessments and an increase in research on BPD may help combat issues with diagnosis.

This research evidenced how online interactions can create peer-to-peer support through mutual sharing of advice, support and encouragement. Peer-to-peer support may be a valuable additional tool in treating mental health difficulties (King and Simmons, 2018; Naslund et al., 2014; Santesteban-Echarri et al., 2017). Peer-to-peer support may bring about social impacts (King and Simmons, 2018). These potential social impacts may have particular relevance for individuals with BPD, who tend to struggle with interpersonal relationships. However, there has been debate about the efficacy of peer-supports. Researchers have found that support groups that use peer support can be a negative experience (Galinsky and Schopler, 1994). A systematic review and meta-analysis conducted by Lyons et al. (2021) found there to be insufficient evidence available on the effectiveness of peer support for mental health issues. Overall, there is a need for more research into the effectiveness of peer support as an intervention before firm conclusions can be drawn (Ali et al., 2015; Lyons et al., 2021).

### 4.4 Influencing factors

All of the vloggers were female and all of them had their appearances complimented. The impressions of the vloggers stories and favourable opinions of the vloggers may have been influenced by the halo effect. Thorndike (1920) first described the halo effect: it is the tendency of people to see positive correlations between traits. As the vloggers physical attractiveness was often complimented, the commenters may have been more likely to rate the vloggers other traits as favourably too. Projection may also have impacted the results, with some commenters using their theoretical knowledge, and feelings and thoughts, and projecting them onto the vloggers (Bazinger and Kühberger, 2012). It is possible that some commenters claiming to be the exact same as the vloggers, were projecting on to them their own feelings and experiences.

Actionability and understandability scores may also have impacted findings. The details on the vlogs and comments (Table 1) show that the vlog with the lowest actionability and understandability scores had longer comments. The vlog may have lacked clarity, and the commenters may have felt the need to assist the vloggers with their explanation. Had the overall PEMAT-A/V actionability and understandability scores been higher for the vlogs, the comments may have been shorter and there also may have been less questions asked.

### 4.5 Strengths and limitations

As far as the authors are aware, this was the first study to analyse comments to YouTube vlogs about living with BPD. By analysing YouTube comments, the study benefitted from lack of researcher interference and a reduction in uneven power dynamics (Fox et al., 2007). As the vlogs gave personal, first-hand accounts, they encouraged interactions between vloggers and commenters, providing a rich and diverse sample (Cunningham and Craig, 2017). The comments gave access to genuine personal reactions (Miller, 2015). Comments can be made at any time and as such, people could comment as soon as they had watched the vlog, even during the vlog. This increased authenticity and spontaneity in responses. A central strength of this research is that it highlights the stigma associated with BPD and the importance of vlogs in countering this stigma, giving hope and encouragement, as well as educating, informing and inspiring.

A key limitation to the study was that all vloggers were female. Even though there are more females than males with BPD (APA, 2013), males can also receive a BPD diagnosis. There is also evidence that females may experience the disorder differently to their male counterparts (Álvarez-Tomás et al., 2019; Masland and Null, 2021). Therefore, male vloggers may have given voice to a different perspective and, as such, elicited different comments.

It is also important to note that the research utilized four vlogs and the comments to these four vlogs are only a snapshot of a population and therefore conclusions are tentative. Another limitation of the study is the inability to validate any of the comments. There is also no way of knowing how many commenters had an actual BPD diagnosis or otherwise, though that does not take away from this study as its aims were to investigate comments from all users, regardless of their mental health status. There was also a limitation brought about by the absence of direct quotes in this final report. The inclusion of quotes may have increased transparency. Instead of direct quotes, the findings were evidenced by paraphrasing the comments. However, to protect the identities of the commenters, paraphrasing is considered best ethical practice when using YouTube comments (Reilly, 2014; Khasawneh et al., 2020).

### 4.6 Areas for future research

There is a need for additional experimental research in this area. Future research could also compare and contrast responses to different vlogs to investigate further the effects of vlogger clarity (vlog understandability and actionability) on the length of comments and commenter disclosures. Researchers may also wish to further explore the differences between males and females vlogging about living with BPD as there may be differences in their experiences and, in turn, commenter responses. Future researchers may investigate the effectiveness and demand for vlogs as a source of support and information about BPD, both before and after the COVID-19 pandemic. Accessing information, support and communities through YouTube may be in increased demand when face-to-face supports are difficult to access (Woloshyn and Savage, 2020).

## 5 Conclusion

YouTube comments are a valuable research tool and are, as yet, underutilized by researchers. BPD is a mental health disorder under-researched, under-diagnosed, misunderstood and stigmatized. As such, research into people's attitudes towards BPD, their opinions and knowledge of the disorder, may help make important changes, inform policies and practice, and ultimately improve the lives of those living with the disorder. This research found personal vlogs on YouTube about BPD to be an important resource for mental health information and advice, having success at destigmatizing BPD and raising awareness. The commenters had been seeking help, support, and understanding, which they received from the vloggers and from their fellow commenters. The evidence in this study underscores the value of studying online environments for the betterment of people experiencing BPD.

## Declaration of competing interest

The authors declare that they have no known competing financial interests or personal relationships that could have appeared to influence the work reported in this paper.

## Figures and Tables

**Table:** 

If disagree	Enter 0
If agree	Enter 1
If not applicable	Enter NA

Title of material: ____________________________________________________ *Name of reviewer*: __________________ *Review date*: ________

**Table:** 

Item #	Item	Response options	Rating
Topic: Content			
1	The material makes its purpose completely evident.	Disagree = 0, agree = 1	

Topic: Word choice & style			
3	The material uses common, everyday language.	Disagree = 0, agree = 1	
4	Medical terms are used only to familiarize audience with the terms. When used, medical terms are defined.	Disagree = 0, agree = 1	
5	The material uses the active voice.	Disagree = 0, agree = 1	

Topic: Organization			
8	The material breaks or “chunks” information into short sections.	Disagree = 0, agree = 1, very short material^i^ = N/A	
9	The material's sections have informative headers.	Disagree = 0, agree = 1, very short material^i^ = N/A	
10	The material presents information in a logical sequence.	Disagree = 0, agree = 1	
11	The material provides a summary.	Disagree = 0, agree = 1, very short material^i^ = N/A	

Topic: Layout & design			
12	The material uses visual cues (e.g., arrows, boxes, bullets, bold, larger font, highlighting) to draw attention to key points.	Disagree = 0, agree = 1, video = N/A	
13	Text on the screen is easy to read.	Disagree = 0, agree = 1, no text or all text is narrated = N/A	
14	The material allows the user to hear the words clearly (e.g., not too fast, not garbled).	Disagree = 0, agree = 1, no narration = N/A	

Topic: Use of visual aids			
18	The material uses illustrations and photographs that are clear and uncluttered.	Disagree = 0, agree = 1, no visual aids = N/A	
19	The material uses simple tables with short and clear row and column headings.	Disagree = 0, agree = 1, no tables = N/A	

*Total points*: _____________.

*Total possible points*: _____________.

Understandability score (%): _____________.

(Total points / Total possible points × 100).

**Table:** 

Item #	Item	Response options	Rating
20	The material clearly identifies at least one action the user can take.	Disagree = 0, agree = 1	
21	The material addresses the user directly when describing actions.	Disagree = 0, agree = 1	
22	The material breaks down any action into manageable, explicit steps.	Disagree = 0, agree = 1	
23	The material explains how to use the charts, graphs, tables, or diagrams to take actions.	Disagree = 0, agree = 1, no charts, graphs, tables, diagrams = N/A	

*Total points*: _____________.

*Total possible points*: _____________.

Actionability score (%): _____________.

(Total points / Total possible points × 100).
